# Effects of a PPAR -gamma receptor agonist and an angiotensin receptor antagonist on aortic contractile responses to alpha receptor agonists in diabetic and/or hypertensive rats

**DOI:** 10.5830/CVJA-2015-080

**Published:** 2016

**Authors:** Ibrahim Tugrul, Turhan Dost, Omer Demir, Filiz Gokalp, Ozlem Oz, Necip Girit, Mustafa Birincioglu

**Affiliations:** Department of Medical Pharmacology, Faculty of Medicine, Adnan Menderes University, Aydin, Turkey; Department of Medical Pharmacology, Faculty of Medicine, Adnan Menderes University, Aydin, Turkey; Department of Medical Pharmacology, Faculty of Medicine, Adnan Menderes University, Aydin, Turkey; Department of Medical Pharmacology, Faculty of Medicine, Adnan Menderes University, Aydin, Turkey; Department of Medical Pharmacology, Faculty of Medicine, Adnan Menderes University, Aydin, Turkey; Department of Medical Pharmacology, Faculty of Medicine, Adnan Menderes University, Aydin, Turkey; Department of Medical Pharmacology, Faculty of Medicine, Adnan Menderes University, Aydin, Turkey

**Keywords:** diabetes, hypertension, pioglitazone, losartan, alpha adrenoceptors

## Abstract

**Aim:**

The aim of this study was to investigate the effects of pioglitazone and losartan pre-treatment on the aortic contractile response to the alpha-1 agonist, phenylephrine, and the alpha-2 agonist, clonidine, in L-NAME-induced hypertensive, STZ-induced diabetic, and hypertensive diabetic rats.

**Methods:**

Male Wistar rats were randomly allocated to four groups: control, diabetic (DM), hypertensive (HT) and hypertensive diabetic (HT + DM) groups. Three weeks after drug application, in vitro dose–response curves to phenylephrine (Phe) (10^-9^–10^-5^ M) and clonidine (Clo) (10^-9^–10^-5^ M) were recorded in aortic rings in the absence (control) and presence of pioglitazone (10 μM) and/or losartan (10 μM).

**Results:**

Pioglitazone and losartan caused a shift to the right in contractile response to phenylephrine in all groups. The sensitivity of the aortic rings to phenylephrine was decreased in the presence of pioglitazone and/or losartan in all groups. The contractile response of clonidine decreased in the presence of pioglitazone and/or losartan in the control, HT and DM groups.

**Conclusion:**

The sensitivity of aortic rings to alpha-1 and alpha-2 adrenoceptors was decreased in the presence of pioglitazone and/or losartan in diabetic and hypertensive rats. Concomitant use of PPAR-gamma agonists, thiazolidinediones, and angiotensin receptor blockers may be effective treatment for diabetes and hypertension.

## Aim

Hypertension and diabetes mellitus are both common diseases worldwide and they co-exist frequently, resulting in significant rates of morbidity and mortality. Diabetes mellitus and hypertension have been identified as risk factors for cardiovascular disease and cause altered vascular responsiveness to several vasoconstrictors and vasodilators.^1^,^2^,^3^ Endotheliumdependent vasodilation is reduced in diabetes, largely due to excessive oxidative stress and the bio-availability of nitric oxide. Endothelium-derived nitric oxide (NO) is a potent endogenous nitrovasodilator and plays a major role in modulation of vascular tone.^4^ NG-nitro-L-arginine methyl ester (L-NAME)- induced hypertension has been one of the most frequently used models of experimental hypertension since 1990.^5^

Thiazolidinediones (TZDs) such as pioglitazone are a class of oral antidiabetic agent that act primarily by decreasing insulin resistance. Drugs in this class act as potent and highly selective agonists for peroxisome proliferator-activated receptor gamma (PPARg).^6^ Pioglitazone repairs blunted endothelium-dependent vasodilatation, protects against oxidative stress and lowers blood pressure.^7^,^8^,^9^,^10^,^11^ The vascular endothelium mediates relaxant responses to a wide range of vasodilators and modulates the constrictor responses to alpha agonists such as phenylephrine and clonidine.

The streptozotocin (STZ)-induced diabetic rat model has been widely used to study changes in vascular reactivity to alpha adrenoceptor agonists.^12^ Hyperglycaemia is likely to modulate physiological responses to angiotensin II and may contribute to the pathogenesis of vascular dysfunction in diabetes.^13^ Angiotensin type 1 receptor (AT_1_R) blockers (ARBs) such as losartan are widely used in the treatment of hypertension.^14^,^15^

It is not clear how concomitant use of medication in the treatment of hypertension and diabetes has effects on vascular contractility. Hence the aim of this study was to investigate the effect of pioglitazone and losartan pre-treatment on the aortic contractile response to the alpha-1 agonist, phenylephrine (Phe), and the alpha-2 agonist, clonidine (Clo), in L-NAME-induced hypertensive, STZ-induced diabetic, and hypertensive diabetic rats.

## Methods

Male Wistar rats (250–300 g) were obtained from the experimental animal centre of Adnan Menderes University and all experiments were performed according to the principles and guidelines of the Adnan Menderes University animal ethics committee. Male Wistar rats were randomly allocated to four groups: a control group (Cont) (n = 15), a diabetic group (DM) (n = 20), a hypertensive group (HT) (n = 20), and a hypertensive diabetic group (HT + DM) (n = 20).

All rats were housed at 22–24°C on a 12-hour dark–light cycle and received food and water (or L-NAME solution in drinking water in the hypertensive groups) ad libitum. Diabetes was induced by a single intraperitoneal injection of 50 mg/kg STZ in the DM group. Hypertension was induced by giving L-NAME (50 mg/kg) in the drinking water for three weeks in the HT group. Hypertension plus diabetes were induced by a single intraperitoneal injection of 50 mg/kg STZ and providing L-NAME (50 mg/kg) in the drinking water for three weeks in the HT + DM group.

Body weights of the treated groups were measured at weekly intervals. In vitro experiments were started three weeks after the drug injections. Systolic blood pressure (SBP) of the rats was measured before the in vitro experiments using the tail-cuff method. Blood was obtained from a tail vein in conscious rats. At least five readings were done at every session and the mean of four values was used to obtain the SBP of each rat. Glucose concentrations were determined using an International Medical Equipment Diabetes Care (IME-DC) blood glucose meter (Oberkotzau, Germany).

## Preparation of aortic rings and in vitro experiments

The rats were anaesthetised with ketamine and xylasine (50 and 5 mg/kg intraperitoneal, respectively). A thoracotomy was performed and the thoracic aorta was removed from the diaphragm to the heart. The aorta was then placed in ice-cold Krebs’ solution where it was cleaned and any adhering fat was removed. The composition of the Krebs’ solution (mmol/l) was 118.0 NaCl; 25.0 NaHCO_3_; 4.7 KCl; 1.2 KH_2_PO_4_; 1.2 MgSO_4_·7H_2_O; 2.5 CaCl_2_; and 10.1 glucose.

The aorta was then cut into small rings (4–5 mm in width). The rings were suspended horizontally between two stainless steel wires and mounted in a 20-ml organ bath filled with warmed (37°C) and oxygenated (95% O2 and 5% CO2) Krebs’ solution. One end of the ring was connected to a force transducer (MAY FDT 05, Commat Ltd. Ankara, Turkey). The rings were equilibrated for 60 min under a resting tension of 2 g with the bath fluids being changed every 15 min. Measurement of the isometric force was recorded on a data-acquisition system (MP 36, Biopac Systems, Inc).

After the equilibration period, the rings were sub-maximally contracted with Phe (10^-7^ M), and the cumulative concentration– response curve to acetylcholine (10^-9^–10^-5^ M) was then obtained to test their contractile capacity. Intact vessels failing to achieve at least 60% relaxation to acetylcholine were assumed to be damaged and were discarded. Cumulative responses to Phe (10^-9^–10^-5^ M) and Clo (10^-9^–10^-5^ M) were recorded in the aortic rings in the absence (control) and presence of pioglitazone (10 μM) and/or losartan (10 μM), which was added to the bathing solution 15 min prior to the contractile responses of Phe or Clo.

Pioglitazone hydrochloride was obtained as a gift sample from Sandoz (Istanbul, Turkey). Streptozotocin, phenylephrine, clonidine, L-NAME and the other chemicals were purchased from Sigma Chemicals. Losartan potassium was purchased from Fluka China (Interlab, Izmir, Turkey).

## Statistical analysis

The results are expressed as mean ± SEM. Statistical evaluation of the data was performed by analysis of variance (ANOVA) and the Student’s t-test. Results were considered significant when p < 0.05. The agonist pD_2_ value (–log EC_50_ ) was calculated from the concentration–response curve by non-linear regression analysis of the curve, using a base-fitting program (Prism, Graphpad).

## Results

STZ-injected animals developed diabetes in the DM and HT + DM groups. In the HT + DM group, five rats died in the first week after the STZ injection. The body weights, blood glucose levels and SBP are shown in Table 1.

**Table 1:** Body weight, blood glucose levels and systolic blood pressure before the in vitro experiments

*Parameters*	*Control group (n = 15)*	*DM group (n = 20)*	*HT group (n = 20)*	*HT+DM group (n = 15)*
Body weight (g)	275.1 ± 6.1	279.1 ± 5.9	309.4 ± 9.5	201.1 ± 7.2^a^
Blood glucose level (mg/dl)	120.3 ± 6.6	371.7 ± 18.1^b^	177.6 ± 15.4	395.4 ± 14.1^b^
Systolic blood pressure (mmHg)	96.4 ± 2.9	155.2 ± 5.2^c^	187.9 ± 3.9^c^	161.5 ± 7.1^c^

Values are expressed as mean ± SEM.

^a^p < 0.05, compared to control group.

^b^p < 0.05, compared to control group. Blood glucose levels > 250 mg/dl (13.88 mmol/l) indicated diabetes.

^c^p < 0.05, compared to control group.

There was a significant increase in blood glucose levels in the STZ-injected groups (DM and HT + DM groups). The daily intake of L-NAME was calculated from the daily water intake and was approximately 21–23 mg/kg/day for the HT and HT + DM groups. There was a significant increase in SBP in the L-NAME-treated groups (HT and HT + DM groups) Table 1.

Phe induced a concentration-dependent contractile response in the aortic rings from all four groups. These curves are shown in (Fig. 1),(Fig. 2),(Fig. 3),(Fig. 4). There was no significant change in maximum contractile response (E_max_) to Phe in all groups due to the presence of pioglitazone and/or losartan; these drugs shifted the contractile response to Phe to the right. The sensitivity of the aortic rings to Phe was however decreased in the presence of pioglitazone and/or losartan in all groups [Table 2. (pD_2_ value)].

**Table 2:** Acute effects of pioglitazone and losartan on vascular sensitivity (pD2) to pheylephrine in segments of thoracic aorta from Wistar rats

**	*Control group pD2 (n = 15)*	*HT group pD2 (n = 7)*	*DM group pD2 (n = 19)*	*HT+DM group pD2 (n = 12)*
Control	7.26 ± 0.08	7.53 ± 0.04	7.29 ± 0.07	7.27 ± 0.07
Pioglitazone	6.80 ± 0.08^a^	7.04 ± 0.07^a^	7.10 ± 0.06^a^	7.23 ± 0.07
Losartan	6.76 ± 0.10^b^	6.95 ± 0.13^b^	7.03 ± 0.06^b^	7.13 ± 0.10
Pioglitazone + losartan	6.61 ± 0.08^c^	6.81 ± 0.08^c^,^d^	6.97 ± 0.05^c^	6.97 ± 0.09^c^,^d^

n is the number of aortic segments in each group. Values are expressed as mean ± SEM.

Cont: control, Pio: pioglitazone, Los: losartan, Pio+Los: pioglitazone + losartan.

Control group: ^a^Cont vs pio (p < 0.001); ^b^Cont vs los (p < 0.001); ^c^Cont vs pio+los (p < 0.001).

HT group: ^a^Cont vs pio (p < 0.001); ^b^Cont vs los (p <0.001); ^c^Cont vs pio+los (p < 0.001); ^d^Pio vs pio+los (p = 0.046).

DM group: ^a^Cont vs pio (p = 0.037); ^b^Cont vs los (p = 0.005); ^c^Cont vs pio+los (p = 0.001).

HT + DM group: ^c^Cont vs pio+los (p = 0.013); ^d^Pio vs pio+los (p = 0.030).

**Figure:**
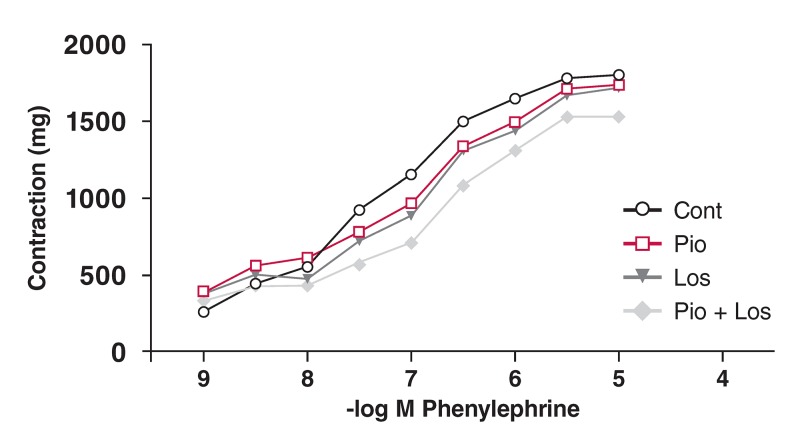
Effects of pioglitazone and losartan on the response of aortic segments to increasing concentrations of phenylephrine in the control group. Cont: control, Pio: pioglitazone, Los: losartan, Pio+Los: pioglitazone + losartan. Values are expressed as mean ± SEM.

**Figure:**
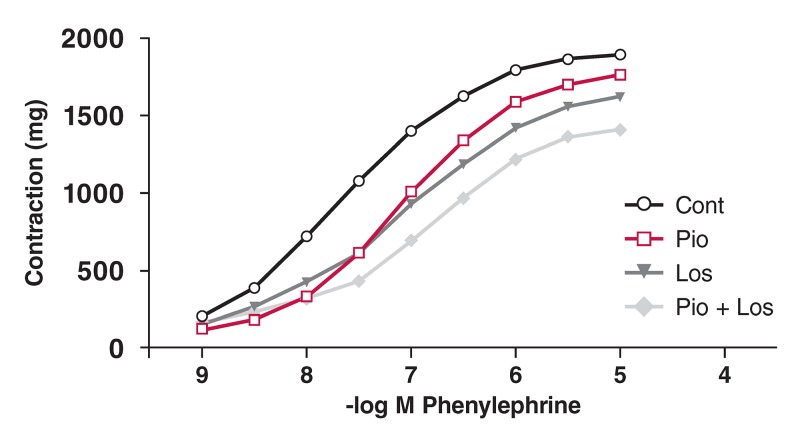
Effects of pioglitazone and losartan on the response of aortic segments to increasing concentrations of phenylephrine in the HT group. Cont: control, Pio: pioglitazone, Los: losartan, Pio+Los: pioglitazone + losartan. Values are expressed as mean ± SEM.

**Figure:**
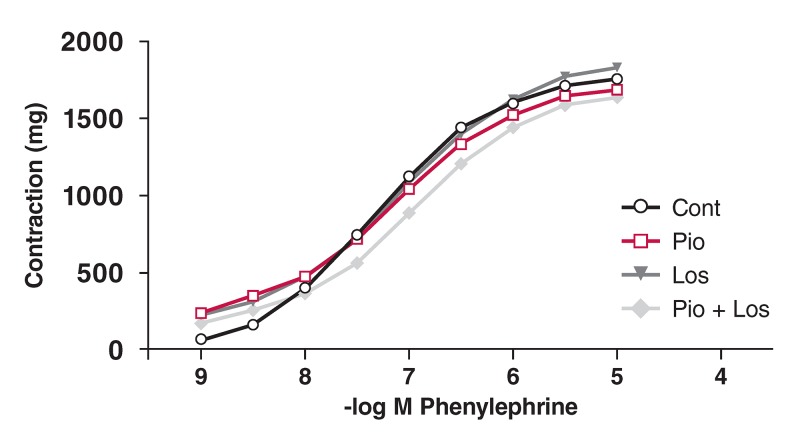
Effects of pioglitazone and losartan on the response of aortic segments to increasing concentrations of phenylephrine in DM group. Cont: control, Pio: pioglitazone, Los: losartan, Pio+Los: pioglitazone + losartan. Values are expressed as mean ± SEM.

**Figure:**
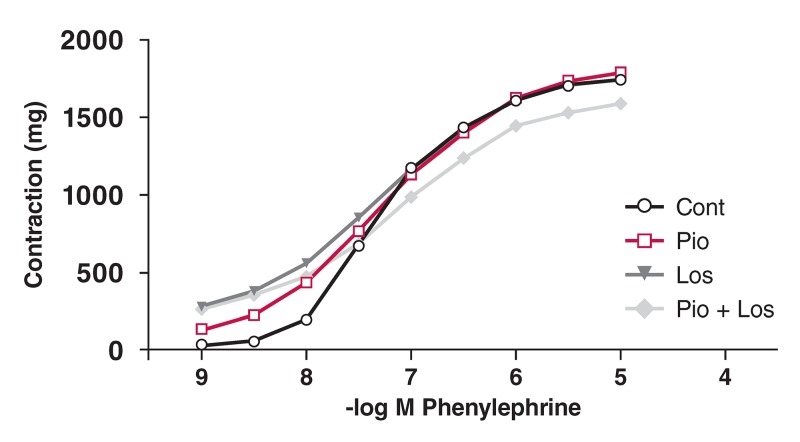
Effects of pioglitazone and losartan on the response of aortic segments to increasing concentrations of phenylephrine in the HT + DM group. Cont: control, Pio: pioglitazone, Los : losartan, Pio+Los: pioglitazone + losartan. Values are expressed as mean ± SEM.

There was significant decrease in maximum contractile response (E_max_) to Clo in the control group due to the presence of pioglitazone and/or losartan (Fig. 5). In the absence of pioglitazone and losartan (control), Clo induced contraction. In the presence of pioglitazone and/or losartan, Clo induced relaxation in the control aortic rings (Fig. 5).

**Figure:**
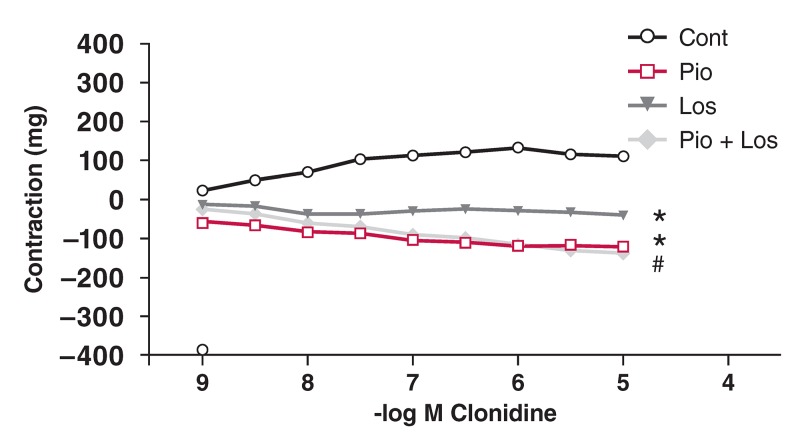
Effects of pioglitazone and losartan on the response of aortic segments to increasing concentrations of clonidine in the control group. Cont: control, Pio: pioglitazone, Los: losartan, Pio+Los: pioglitazone + losartan. Values are expressed as mean ± SEM (n = 14). *Cont vs Pio (p = 0.001); *Cont vs Los (p = 0.011); #Cont vs Pio+Los (p < 0.001).

In the HT group, Clo did not cause relaxation. The contractile response to Clo was decreased in the presence of pioglitazone and/or losartan (Fig. 6). In the DM group, the contractile response to Clo was significantly decreased in the presence of pioglitazone and losartan, but not in the presence of either pioglitazone or losartan alone (Fig. 7). In the HT + DM group, the decrease in contractile response to Clo was not significant in the presence of pioglitazone and losartan (Fig. 8).

**Figure:**
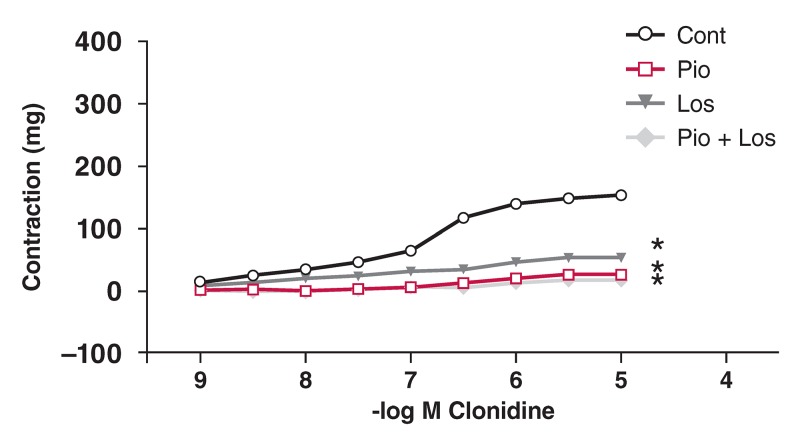
Effects of pioglitazone and losartan on the response of aortic segments to increasing concentrations of clonidine in the HT group. Cont: control, Pio: pioglitazone, Los: losartan, Pio+Los: pioglitazone + losartan. Values are expressed as mean ± SEM (n = 5). *Cont vs Pio (p = 0.004); *Cont Clo vs Los (p = 0.014); *Cont Clo vs Pio+Los (p = 0.001).

**Figure:**
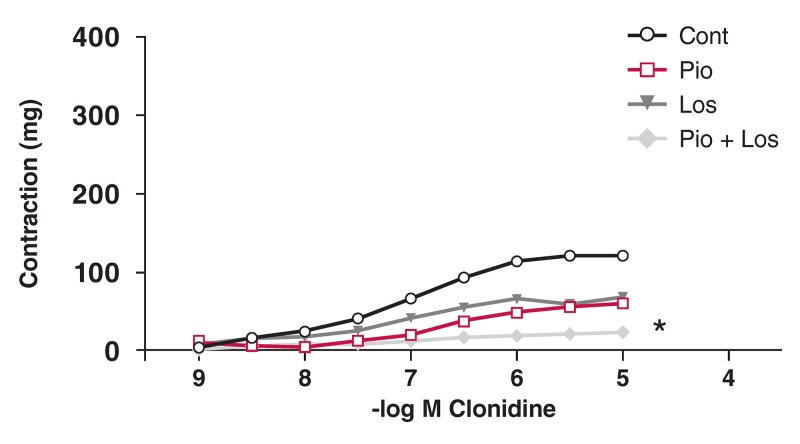
Effects of pioglitazone and losartan on the response of aortic segments to increasing concentrations of clonidine in DM group. Cont: control, Pio: pioglitazone, Los: losartan, Pio+Los: pioglitazone + losartan. Values are expressed as mean ± SEM (n = 16). *Cont vs Pio+Los (p = 0.005).

**Figure:**
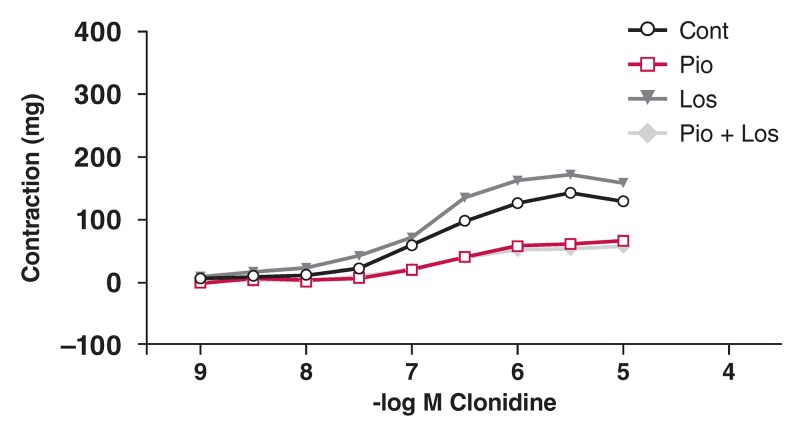
Effects of pioglitazone and losartan on the response of aortic segments to increasing concentrations of clonidine in the HT + DM group. Cont: control, Pio: pioglitazone, Los: losartan, Pio+Los: pioglitazone + losartan. Values are expressed as mean ± SEM (n = 13).

## Discussion

This study investigated the effects of pioglitazone and losartan on aortic contractile responses to alpha adrenoceptors in diabetic and/or hypertensive rats. We examined the effects of pioglitazone and losartan on vascular contractility in control, L-NAME-induced hypertensive, STZ-induced diabetic, and hypertensive diabetic rats. The major findings of this study were that pre-treatment of rat aortic rings with pioglitazone (10 μM) and/or losartan (10 μM) decreased the sensitivity of the contractile responses to phenylephrine and decreased the maximum clonidine contraction.

Various authors have reported on the blood pressurelowering effects of PPAR-gamma agonists such as pioglitazone in rats and monkeys, and in patients with type 2 diabetes and hypertension.^9^,^6^,^17^,^18^ Majithiya et al. noted an increase in SBP in STZ-induced (55 mg/kg, intravenous) diabetic Sprague-Dawley rats, and also reported that pioglitazone administration to these rats lowered their blood pressure.^10^ Diep et al. showed that treatment with pioglitazone (10 mg/kg/day) or rosiglitazone (5 mg/kg/day) for seven days attenuated the development of hypertension, improved endothelial dysfunction induced by angiotensin II infusion, and corrected vascular structural abnormalities.^19^

Nomura and co-workers reported their findings regarding the effect of pioglitazone on the contractility of isolated blood vessels.^20^ Buchanan and colleagues showed that the addition of pioglitazone to vascular preparations decreased KCl- and norepinephrine-induced vasoconstriction in vitro.^11^ Accordng to Majithiya and co-workers, administration of pioglitazone for four weeks restored elevated blood pressure to normal, reduced the enhanced contractility to phenylephrine, and restored acetyl choline-induced relaxation.^10^

The endothelium is involved in the beneficial vascular action of the glitazones.^21^ Various authors have shown that pioglitazone directly dilates blood vessels by blocking the calcium channels.^11^,^22^ It has been reported that a decrease in blood pressure due to pioglitazone is due to direct dilation of the vascular smooth muscles by blocking the calcium channels or reducing total peripheral resistance.^11^,^22^,^23^

In vivo PPAR-alpha and -gamma agonists have been shown to reduce superoxide generation, restore endothelial dysfunction and improve vasorelaxation to acetyl choline in the aorta of diabetic rats.^10^,^24^ Majithiya and colleagues reported that treatment with pioglitazone reduced blood pressure, reduced oxidative stress and restored endothelial function in STZ-induced diabetic rats. The fact that pioglitazone reduced oxidative stress may have been a cause of the reduction in blood pressure.

The protective effect of pioglitazone against oxidative stress may prevent the breakdown of NO, which may improve vascular function. Similar observations were made by Bagi and co-workers that pioglitazone increased NO bio-availability and reduced oxidative stress in coronary arterioles of mice with type 2 diabetes.^25^ Matsumoto and colleagues reported that chronic treatment with pioglitazone restored impaired NO-mediated, endotheliumdependent relaxation in diabetic rat aortae.^26^ It has been shown that reduction in blood pressure in the case of STZ-induced diabetic rats was NO mediated.^4^ Calnek and co-workers reported that PPARgamma agonists increased NO bioavailability in cultured cells.^27^

Pioglitazone was shown to directly induce a relaxation of rat aortae pre-contracted with phenylephrine, which was inhibited by L-NAME.^10^ Similarly, indomethacin-treated vessels incubated with pioglitazone markedly reduced the phenylephrine contractions.^3^ Although most researchers agree that the sensitivity to phenylephrine was unchanged during the early stage of diabetes (up to 12 weeks in STZ-induced diabetic rats), they disagree on the response to phenylephrine. Agrawal and McNeill reported an increase in contractility in response to phenylephrine,^28^ Pfaffman and co-workers reported a decrease,^29^ and Scarborough and Carrier and White and Carrier reported no change.^30^,^31^ In contrast, studies that extended the diabetic duration up to 43–52 weeks have demonstrated a consistent increase in sensitivity to noradrenaline in rat aortae^32^ and mesenteric arteries^33^ from STZ-induced diabetic rats.

In our study, we suggest that our diabetic rats did not have enough time to develop a sufficiently severe degree of vascular dysfunction to manifest an effect to phenylephrine. From our results, acute pioglitazone/losartan pre-treatment did not significantly change the maximum contractile responses to phenylephrine in the control, diabetic or hypertensive rats.

We attempted to determine whether these drugs affected the endothelial modulatory responses to vasoconstriction produced by phenylephrine. Sensitivity of the aortic rings to phenylephrine was decreased in the presence of pioglitazone and/or losartan. The glitazones have been shown by Asano et al. to decrease smooth muscle cell contractility,^34^ and by Dormandy et al. to cause improvement in vascular function.^35^ We believe, however, that the blunted adrenergic responses observed in the presence of glitazones were mediated by the action of these drugs on the endothelial cells, since the effect disappeared when the endothelium was removed in a study Mendizabal and co-workers.^21^

## Conclusion

In this study, in vitro experiments were carried out to investigate the direct effect of pioglitazone and/or losartan on aortic rings of control, diabetic, hypertensive and hypertensive diabetic rats. Our results demonstrate that vascular sensitivity to an alpha adrenoceptor agonist was decreased in the presence of pioglitazone and/or losartan in diabetic and/or hypertensive rat aortic rings. We postulate that these results explain at least in part the beneficial effects of pioglitazone and losartan for hypertension and diabetes. The mechanism of action of pioglitazone and losartan to improve vascular reactivity may be as a result of intracellular protection from oxygen free radicals. Our findings suggest a possible beneficial combination of thiazolidinediones and angiotensin receptor blockers for treatment of diabetes and hypertension.

Further studies are required to elucidate the effects of pioglitazone and losartan on alpha receptors and on the mediators of NO metabolism. It is also remains unclear how pioglitazone and losartan inhibited alpha-2 receptor activities in our rat aortic rings. Further investigation is needed to clarify these underlying mechanisms.
